# Associations between infant fungal and bacterial dysbiosis and childhood atopic wheeze in a nonindustrialized setting

**DOI:** 10.1016/j.jaci.2017.08.041

**Published:** 2018-08

**Authors:** Marie-Claire Arrieta, Andrea Arévalo, Leah Stiemsma, Pedro Dimitriu, Martha E. Chico, Sofia Loor, Maritza Vaca, Rozlyn C.T. Boutin, Evan Morien, Mingliang Jin, Stuart E. Turvey, Jens Walter, Laura Wegener Parfrey, Philip J. Cooper, Brett Finlay

**Affiliations:** a Michael Smith Laboratories and the Department of Microbiology and Immunology, University of British Columbia, Vancouver, British Columbia, Canada; g Department of Microbiology and Immunology, University of British Columbia, Vancouver, British Columbia, Canada; i Departments of Zoology and Botany, University of British Columbia, Vancouver, British Columbia, Canada; l Department of Biochemistry and Molecular Biology, University of British Columbia, Vancouver, British Columbia, Canada; b Department of Physiology and Pharmacology, University of Calgary, Calgary, Alberta, Canada; c Department of Pediatrics, University of Calgary, Calgary, Alberta, Canada; d Facultad de Ciencias Medicas, de la Salud y la Vida, Universidad Internacional del Ecuador, Quito, Ecuador; e Department of Pediatrics, BC Children's Hospital, University of British Columbia, Vancouver, British Columbia, Canada; f Department of Epidemiology, Fielding School of Public Health, University of California Los Angeles, Los Angeles, Calif; h Fundación Ecuatoriana Para Investigación en Salud, Quito, Ecuador; j Department of Agricultural, Food and Nutritional Sciences, University of Alberta, Edmonton, Alberta, Canada; k Institute of Infection and Immunity, St George's University of London, London, United Kingdom

**Keywords:** Asthma, atopy, wheeze, gut microbiome, mycobiome, short-chain fatty acids, nonindustrialized setting, AW, Atopic patient with wheeze, CHILD, Canadian Healthy Infant Longitudinal Development, MaAsLin, Multivariate association with linear models, MED, Minimum Entropy Decomposition, OTU, Operational taxonomic unit, PICRUSt, Phylogenetic Investigation of Communities by Reconstruction of Unobserved States, qPCR, Quantitative PCR, SCFA, Short-chain fatty acid

## Abstract

**Background:**

Asthma is the most prevalent chronic disease of childhood. Recently, we identified a critical window early in the life of both mice and Canadian infants during which gut microbial changes (dysbiosis) affect asthma development. Given geographic differences in human gut microbiota worldwide, we studied the effects of gut microbial dysbiosis on atopic wheeze in a population living in a distinct developing world environment.

**Objective:**

We sought to determine whether microbial alterations in early infancy are associated with the development of atopic wheeze in a nonindustrialized setting.

**Methods:**

We conducted a case-control study nested within a birth cohort from rural Ecuador in which we identified 27 children with atopic wheeze and 70 healthy control subjects at 5 years of age. We analyzed bacterial and eukaryotic gut microbiota in stool samples collected at 3 months of age using 16S and 18S sequencing. Bacterial metagenomes were predicted from 16S rRNA data by using Phylogenetic Investigation of Communities by Reconstruction of Unobserved States and categorized by function with Kyoto Encyclopedia of Genes and Genomes ontology. Concentrations of fecal short-chain fatty acids were determined by using gas chromatography.

**Results:**

As previously observed in Canadian infants, microbial dysbiosis at 3 months of age was associated with later development of atopic wheeze. However, the dysbiosis in Ecuadorian babies involved different bacterial taxa, was more pronounced, and also involved several fungal taxa. Predicted metagenomic analysis emphasized significant dysbiosis-associated differences in genes involved in carbohydrate and taurine metabolism. Levels of the fecal short-chain fatty acids acetate and caproate were reduced and increased, respectively, in the 3-month stool samples of children who went on to have atopic wheeze.

**Conclusions:**

Our findings support the importance of fungal and bacterial microbiota during the first 100 days of life on the development of atopic wheeze and provide additional support for considering modulation of the gut microbiome as a primary asthma prevention strategy.

## Graphical abstract

**Figure:**
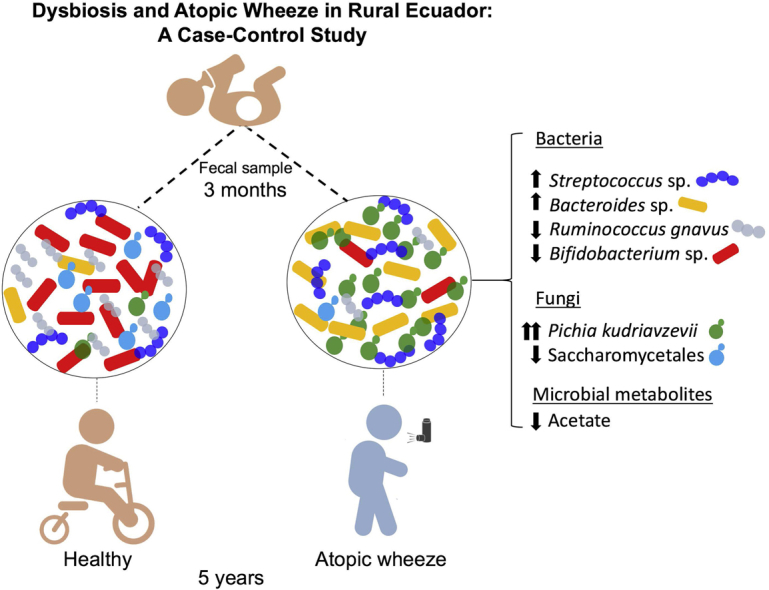

Asthma is a disease of the airways that affects more than 235 million persons worldwide,^1^ causing an enormous burden of disease and high economic costs to societies. Asthma has increased dramatically in incidence in industrialized countries since the 1960s, making it the most common chronic disease of childhood.^1^, ^2^ The increase in prevalence has been attributed primarily to environmental alterations rather than changes in genetic susceptibility.^2^ Epidemiologic studies have revealed several potentially protective environmental factors, such as growing up on a farm, vaginal birth, breast-feeding, the presence of household pets, birth order, and number of children, as well an increased risk of asthma being associated with antibiotic use during late pregnancy and the first year of life.^3^ The nature of these exposures has led many to hypothesize that microbial alterations (dysbiosis) driven by these factors and leading to modulation of the infant immune response are causally related to the increase in asthma and other atopic diseases.

Previously, we provided strong support for this hypothesis in mice^4^, ^5^, ^6^ and human subjects,^7^, ^8^ in which we found a critical window early in life during which changes in the gut microbiome are influential in immune dysregulation and can lead to atopy and asthma.^9^ Importantly, we identified specific bacterial taxa associated with reduced asthma risk in a prospective longitudinal cohort study of Canadian babies (Canadian Healthy Infant Longitudinal Development [CHILD] study).^10^ These microbial taxa were not only associated with disease but also implicated causally in ameliorating experimental asthma in a murine model.^7^ A more recent microbiome study in a US pediatric cohort also showed bacterial dysbiosis in 1-month-old infants at high risk of asthma.^11^ Interestingly, this study also measured the fecal fungal community and found several fungal taxa also involved in the gut dysbiosis associated with asthma.^11^ Traditionally not included in microbiome studies, fungi are important members of the gut microbiome, especially in the first months of life, when fungi are present at a much higher diversity than later in life.^11^

Because asthma rates continue to climb in affluent countries, the less developed regions of the world are now also experiencing increases in asthma prevalence, particularly urban and urbanizing populations. The International Study of Asthma and Allergies in Childhood^12^, ^13^ reported asthma prevalence worldwide and showed that some of the highest rates were in Latin American countries, such as Peru, Brazil, and Costa Rica. Although this has been taken as evidence against the hygiene hypothesis, rural populations within these countries have lower rates of asthma than urban populations, and rural-to-urban migration and modernization can contribute to increases in asthma prevalence.^14^ Thus changes in microbial exposure driven by urbanization might help explain regional differences in asthma within Latin America.

The intestinal microbiome differs by geographic location, which is likely explained by differences in diet, lifestyle, and environmental exposures.^15^ Not surprisingly, the limited data available on the microbiome of Latin Americans show notable differences between the gut microbiome from this part of the world and North American or European populations.^15^, ^16^ Given what we have reported previously in Canadian infants, we explored whether similar patterns can be observed in a geographically distinct population with similar reported rates of asthma prevalence to Canada (approximately 10% of the population).^17^

To do this, we completed a case-control study nested within a birth cohort from a rural district in a tropical coastal region of Ecuador, in which we selected children who had atopic wheeze at 5 years of age and healthy control subjects. We compared the bacterial and fungal microbiomes and levels of short-chain fatty acids (SCFAs) as a measure of bacterial carbohydrate metabolism in fecal samples collected at 3 months and contrasted our findings with those obtained from a Canadian cohort. Here we show that bacterial and fungal dysbiosis can be detected in early life in babies who go onto have atopic wheeze in later childhood in a nonindustrialized setting.

## Methods

### Study design

We conducted a case-control study nested within the ECUAVIDA birth cohort study designed to study prospectively the effects of early-life parasite infections on the development of atopy and allergic diseases. The cohort of 2404 newborns was recruited between 2006 and 2009 in the public hospital serving the rural district of Quinindé, Esmeraldas Province, a tropical region of coastal Ecuador. Detailed information for the cohort is provided elsewhere.^18^ Cases and control subjects were selected from children with a stool sample collected at 3 months of age. As outlined in more detail below, cases were defined as children with maternally reported wheeze in the previous 12 months at 5 years of age and with evidence of atopy based on a positive skin prick test response. Healthy control subjects were a random sample of children with no previous history of wheeze and no evidence of atopy at 5 years of age selected by using the sample option in Stata 11 software (StataCorp, College Station, Tex). The study protocols were approved by the Ethics Committees of the Hospital Pedro Vicente Maldonado and Universidad San Francisco, Quito, Ecuador. The study is registered as an observational study (ISRCTN41239086). Informed written consent was obtained from the child's mother.

### Data collection and study procedures

Data on wheezing were collected by using maternal questionnaires administered by a trained physician (M.E.C. and M.V.). Wheeze was defined as any episode of reported wheeze during the previous 12 months. Other data were collected by using periodic questionnaires administered during the first 2 weeks of life and then at 7 and 13 months. Blood was collected at 7 and 13 months and analyzed for eosinophil counts by using standard methods. Stool samples were collected from the child at 3 months and analyzed by using microscopic methods, as previously described.^19^ An aliquot was stored at −80°C until analysis.

Atopy was measured based on skin prick test reactivity at 5 years of age using 9 common allergen extracts (Greer Laboratories, Lenoir, NC): *Dermatophagoides pteronyssinus*/*farinae* mix, American cockroach *(Periplaneta americana)*, cat, dog, grass pollen (9 southern grass mix), fungi, egg, milk, and peanut, with positive histamine and negative saline controls. A positive reaction was defined as a mean wheal diameter of at least 3 mm greater than that elicited by the saline control, and a positive skin prick test response was defined as a positive reaction to any of the allergens tested. No children were taking antihistamines at the time of skin testing.

### Microbial community analysis

#### Extraction and amplicon preparation

DNA was extracted from approximately 50 mg of stool. Samples were mechanically lysed by using Mo Bio dry bead tubes (Mo Bio Laboratories, San Diego, Calif) and the FastPrep Homogenizer (FastPrep Instrument; MP Biomedicals, Santa Ana, Calif) before DNA extraction with the Qiagen DNA Stool Mini Kit (Qiagen, Hilden, Germany).

All samples were amplified by means of PCR in triplicate by using barcoded primer pairs flanking the V4 region of the 16S gene with the forward primer 5′-GTGCCAGCMGCCGCGGTAA–3′ and the reverse primer 5-GGACTACHVGGGTWTCTAAT-3′, as previously described.^20^

##### 16S

Each 50 μL of PCR contained 22 μL of water, 25 μL of TopTaq Master Mix, 0.5 μL of each forward and reverse barcoded primer, and 2 μL of template DNA. The PCR program consisted of an initial DNA denaturation step at 95°C for 5 minutes, 25 cycles of DNA denaturation at 95°C for 1 minute, an annealing step at 50°C for 1 minute, an elongation step at 72°C for 1 minute, and a final elongation step at 72°C for 7 minutes. Controls without template DNA were included to ensure that no contamination occurred. Amplicons were run on a 2% agarose gel to ensure adequate amplification. Amplicons displaying bands at approximately 250 bp were purified with the Illustra GFX PCR DNA Purification Kit (GE Healthcare, Fairfield, Conn). Purified samples were diluted 1:50 and quantified with PicoGreen (Invitrogen, Carlsbad, Calif) in the TECAN M200 (excitation at 480 nm and emission at 520 nm).

##### 18S

DNA was sent to Integrated Microbiome Resource at Dalhousie University (Halifax, Nova Scotia, Canada) for amplification and sequencing. The 18S gene was amplified with the primers E572F (5′-CYGCGGTAATTCCAGCTC-3′) and E1009R (5′-AYGGTATCTRATCRTCTTYG-3′), and the reaction included a PNA blocking primer to reduce amplification of mammalian sequences (5′-TCTTAATCATGGCCTCAGTT-3′). Amplification was done in duplicate, with one reaction using undiluted DNA and one reaction using DNA diluted 1:10 in PCR water. Amplification was done according to previously described protocols.^21^

#### Sequencing

##### 16S

Each PCR pool was analyzed on the Agilent Bioanalyzer (Agilent Technologies, Santa Clara, Calif) by using the High Sensitivity DS DNA assay to determine approximate library fragment size and to verify library integrity. Pooled library concentrations are determined by using the KAPA Library Quantification Kit (Illumina, San Diego, Calif). Library pools are diluted to 4 nmol/L and denatured into single strands by using fresh 0.2 N NaOH. The final library loading concentration is 8 pmol/L, with an additional PhiX spike-in of 20%. The amplicon library is sequenced on the MiSeq by using the 8MiSeq 500 Cycle V2 Reagent Kit (250 × 2).

##### 18S

PCR products were visualized on E-Gels (Thermo Fisher Scientific, Waltham, Mass). The PCR product was quantified with the Invitrogen Qubit with PicoGreen and pooled at equal concentrations, according to the method of Comeau et al.^21^ PhiX was spiked in at 5%, and the resulting library was sequenced at Dalhousie University on the Illumina MiSeq with the 8MiSeq 500 Cycle V2 Reagent Kit (250 × 2).

#### Bioinformatics

Sequences were preprocessed, denoised, and quality filtered by size by using Mothur MiSeq SOP (16S)^22^ or QIIME (18S).^23^

Demultiplexed reads were trimmed to a uniform length of 250 bp by using the FastX Toolkit (http://hannonlab.cshl.edu/fastx_toolkit/) and clustered into operational taxonomic units (OTUs) by using the Minimum Entropy Decomposition (MED) method,^24^ as implemented in the Oligotyping microbial analysis software package.^25^ MED is used to perform *de novo* taxonomic clustering with Shannon entropy to separate biologically meaningful patterns of nucleotide diversity from sequencing noise; processed data are partitioned into phylogenetically homogeneous units (MED nodes) for downstream bacterial diversity analyses. This analysis was carried out with the minimum substantive abundance parameter (-M) set at 250 reads. All other parameters were run with default settings; the maximum variation allowed per node (-V) was automatically set at 3 nucleotides.

Representative sequences were classified by clustering against the Greengenes database at 97% similarity (16S)^26^ or SILVA123 at 99% similarity (18S).^27^ The 16S data set was filtered to remove mitochondria and chloroplast sequences. The 18S data set was filtered to remove mammalian sequences, plants, and all OTUs present in fewer than 3 samples. After filtering, a cutoff of 1000 reads per sample was applied. All 16S samples passed the cutoff. In the 18S data set many samples had fewer than 1000 sequences per sample after filtering. Thus 10 samples from atopic patients with wheeze (AWs) and 22 control samples were retained for statistical analysis of the 18S data set. It is important to apply this sequencing cutoff because a low number of sequences might be a consequence of amplification or sequencing artifacts that might not reflect a real microbial community and will not provide enough statistical power for analysis. Singleton OTUs and any OTUs present less than 3 times among all samples were removed from both the 16S and 18S analyses. We constructed a phylogenetic tree for *Pichia* species by first extracting sequences from the aligned 99% SILVA 123 data set that corresponded to the genus *Pichia* or to the family Pichiaceae with RAxML.^28^ The short Illumina reads were then aligned to the SILVA 123 data set by using PyNAST^29^ and placed in the reference *Pichia* species tree by using the maximum likelihood RAxML evolutionary placement algorithm.^30^

### Quantitative PCR

Quantitative PCR (qPCR) was performed on genomic DNA isolated from stool samples by using the QIAamp Fast DNA Stool Mini Kit (Qiagen) to validate the high-throughput sequencing results. After extraction, all DNA concentrations were measured with the Quant-iT PicoGreen dsDNA Assay Kit (Thermo Fisher Scientific), and template DNA used in the qPCR reactions was obtained from stocks normalized to 1 or 0.2 ng/μL (samples 2, 7, 11, 12, 14, 28, 54, 56, 59, 61, 66, 67, 71, 73, 79, and 93) and diluted as necessary.

*Pichia kudriavzevii*–specific primers targeting the 18S rRNA gene of this organism (forward: 5′-CAT TCC GGG GGG CAT GCC T-3′ and reverse: 5′-GGC CAG CTT CGC TCC CTT TCA-3′) and fungi-specific 18S primers (FR1: 5′-AIC CAT TCA ATC GGT AIT-3′ and FF390: 5′-CGA TAA CGA ACG AGA CCT-3′)^31^ were used to measure the absolute abundance of *P kudriavzevii* and fungal 18S gene copies, respectively, by using the absolute quantification method. Primers for *P kudriavzevii* were designed by using CLUSTALW and National Center for Biotechnology Information Primer BLAST software, and their specificity for *P kudriavzevii* was verified by sequencing the amplicons obtained from a PCR reaction done with genomic DNA combined from 5 chosen subjects shown to be enriched in *P kudriavzevii* based on the sequencing data.

#### qPCR standard curve generation

**Fig E1:**
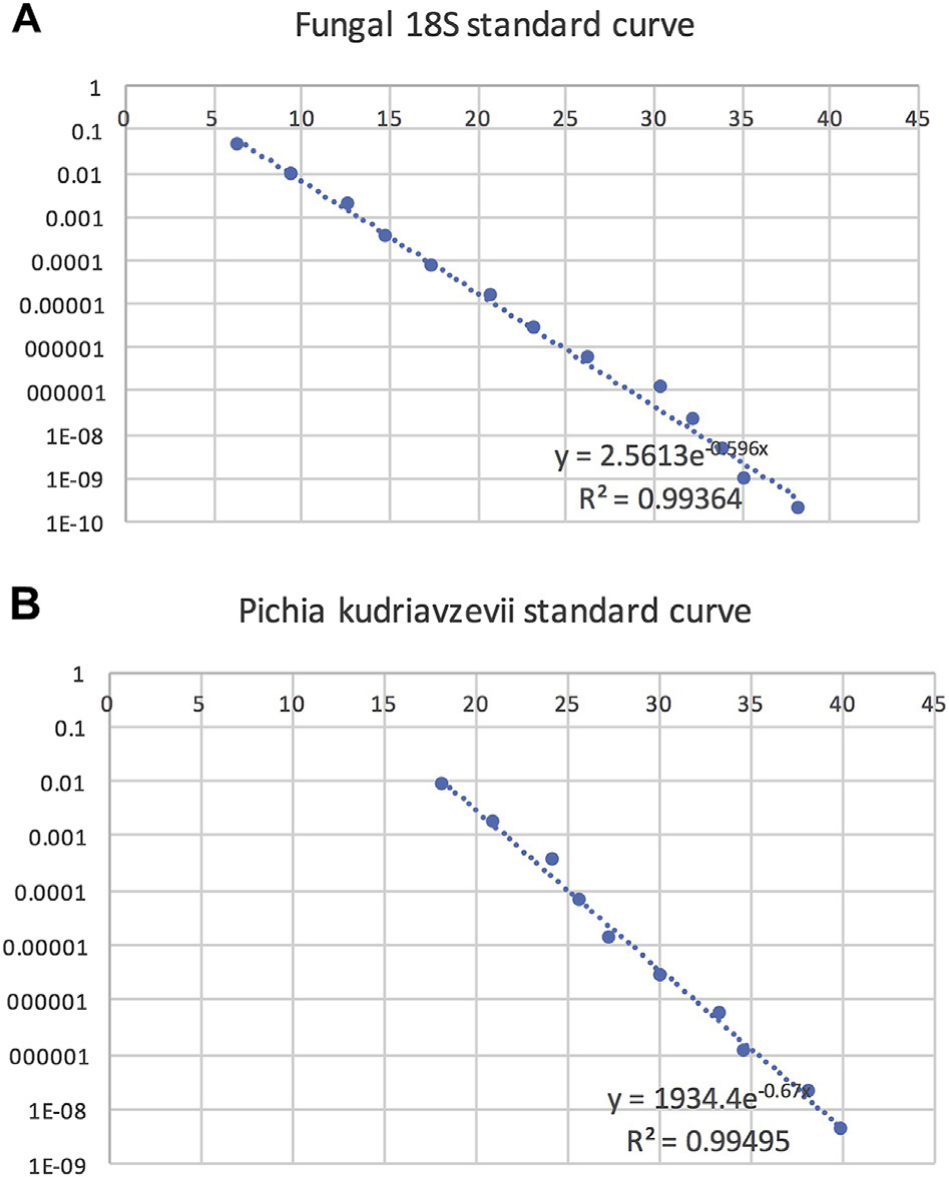
Standard curves for fungus-specific 18S **(A)** and *P kudriavzevii***(B)** qPCR reactions. Standard curves were generated by using 1:5 dilutions of a 1 ng/μL stock of 18S amplicons obtained from PCR reactions done with fungus-specific 18S primers (FR1 and FF390) and purified *P kudriavzevii* template DNA (see the Methods section). DNA concentrations are denoted in logarithmic scale *(y-axis)* in relation to PCR cycle number *(x-axis)*. Exponential trend lines and equations, as well as *R*^*2*^ values, are included in each graph.

18S standard curves (see Fig E1 in this article's Online Repository at www.jacionline.org) were generated by using 1:5 dilutions of a 1 ng/μL stock of 18S amplicons obtained from PCR reactions done with the fungi-specific 18S primers (FR1 and FF390) and purified *P kudriavzevii* template DNA. Template DNA was extracted by using the YeaStar Genomic DNA Kit (Zymo Research, Irvine, Calif) from pure liquid cultures (ATCC 6258) grown for 24 hours at 30°C in YPD media. A *P kudriavzevii* standard curve was generated by performing 1:5 dilutions of a 1 ng/μL stock of the same purified *P kudriavzevii* DNA (see Fig E1).

#### qPCR reactions

All qPCR reactions were performed in duplicate on all stool samples for which there was sufficient DNA (n = 95; samples 3 and 19 were not included) by using the 7500 Fast Real-Time System (Applied Biosystems, Foster City, Calif). Each 10-μL qPCR reaction done with the *P kudriavzevii*–specific primers contained 5 μL of iQ SYBR Green Supermix (Bio-Rad Laboratories, Hercules, Calif), 0.5 μL of each of the forward and reverse primers, and 4 μL of 0.2 ng/μL template DNA. The qPCR program consisted of 2 initial steps of 20 seconds at 55°C and 10 minutes at 95°C, followed by 40 cycles of 30 seconds at 95°C and 60 seconds at 60°C and a final cycle of 95°C at 15 seconds, 60°C at 1 minute, 95°C at 15 seconds, and 60°C at 15 seconds.

Each 20-μL reaction used for 18S rDNA quantitation contained 10 μL of iQ SYBR Green Supermix, 2.5 μL of each of the FR1 and FF390 primers, 3 μL of nuclease-free water, and 2 μL of 1 ng/μL genomic DNA. The qPCR program consisted of an initial step at 95°C for 10 minutes, followed by 40 cycles of 15 seconds at 95°C, 30 seconds at 50°C, and 60 seconds at 70°C, with a final cycle of 95°C at 15 seconds, 60°C at 1 minute, 95°C at 15 seconds, and 60°C at 15 seconds.

### Phylogenetic Investigation of Communities by Reconstruction of Unobserved States

We used Phylogenetic Investigation of Communities by Reconstruction of Unobserved States (PICRUSt)^32^ to generate a profile of putative functions (through metagenomic prediction) from the 16S rRNA OTU data. Predicted metagenomes from all samples were categorized by function at Kyoto Encyclopedia of Genes and Genome orthology level 3. To test for significant differences in functional category abundances between the AW and control samples, we used the Welch *t* test implementation of STAMP.^33^ We also tested for differentially abundant predicted genes with DESeq2^34^ under default settings. DESeq2 estimates variance and mean dependence in count data from high-throughput data sets and tests for a differential expression based on a model using negative binomial distribution. The test statistics' *P* values were adjusted for multiple testing by using the procedure of Benjamini and Hochberg (false discovery rate threshold, 5%).

### SCFA analysis

Infants' fecal samples were combined with 25% phosphoric acid, vortexed, and centrifuged until a clear supernatant was obtained. Supernatants were submitted for gas chromatography to the Department of Agricultural, Food and Nutritional Science of the University of Alberta (Edmonton, Alberta, Canada). Samples were analyzed, as previously described,^35^ with modifications. Briefly, samples were combined with 4-methyl-valeric acid as an internal standard, and 0.2 mL was injected into the Bruker SCION 456 gas chromatograph by using a Stabilwax-DA 30-m × 0.53-mm × 0.5-μm column (Restek, Bellefonte, Pa). A standard solution containing acetic acid, proprionic acid, isobutyric acid, butyric acid, isovaleric acid, valeric acid, and caproic acid, combined with internal standard, was injected in every run.

Programmable temperature vaporization injector and flame ionization detector temperatures were held at 250°C for the entire run. The oven was started at 80°C and immediately ramped to 210°C at 45°C/min, where it was held for 5.11 minutes. Total run time was 8.00 minutes. Helium was used at a constant flow of 20.00 mL/min. Sample concentrations were normalized to the wet weight of feces.

### Statistical analysis

Differences in frequencies in categorical and continuous variables between cases and control subjects were evaluated by using χ^2^ and Mann-Whitney tests, respectively. The Fisher exact test was used for the reduced sample available for fungal sequencing analysis. We assessed fecal microbial diversity and relative abundance of bacterial and fungal taxa by using phyloseq,^36^ along with additional R-based computational tools^37^, ^38^, ^39^, ^40^, ^41^, ^42^ in Rstudio software. Principal coordinates analysis was conducted by using phyloseq (Bray-Curtis as distance metric) and statistically confirmed with permutational multivariate ANOVA. The Chao1 index was calculated by using phyloseq and statistically confirmed with the Mann-Whitney test (GraphPad Prism software, version 5c; GraphPad Software, La Jolla, Calif). The R packages DESeq2^34^ and multivariate association with linear models (MaAsLin)^43^ were used to calculate differentially abundant OTUs between the control and AW samples. From the available metadata, the following covariates were forced into the MaAsLin model based on significant associations with the 5-year AW phenotype and on previously reported associations with asthma or with microbiome shifts: antibiotic use during pregnancy or the first year of life, duration of antibiotic use during pregnancy or the first year of life, type of delivery, household potable water, number of respiratory tract infections during the first year of life, eosinophilia at 7 months, and number of diarrheal episodes during the first year of life. All ecological measurements, except for the Chao1 index, were performed on the 100 most abundant OTUs after normalizing OTUs for relative abundance, which represent 98.14% and 99.83% of total OTUs in the 16S and 18S data sets, respectively.

## Results

### Study subject characteristics: AWs and control subjects

From 1066 stool samples collected from infants in the ECUAVIDA cohort at 3 months of age with follow-up data at 5 years of age (2090), we identified 27 AWs and a random sample of 70 healthy control subjects with no history of wheeze and no atopy at 5 years. The required sample size for microbiome analysis was determined by using MetSizeR, a software package designed to estimate sample size for high-throughput experiments. Assuming at least 100 parameters measured and that 10% would differ significantly between cases and control subjects, a sample size of 24 was required to maintain a false discovery rate of less than 0.05. Characteristics of AWs and control subjects are shown in Table I. Most variables were distributed similarly between the 2 groups with the exceptions of mode of delivery (caesarean delivery: 37% for AWs vs 16% for control subjects, *P* = .022) and number of documented acute respiratory tract infections, which were more frequent in AWs compared with control subjects (*P* = .011). There were no significant differences with respect to baseline characteristics between AWs included in this analysis and those not included for lack of a 3-month stool sample (see Table E1 in this article's Online Repository at www.jacionline.org).

**Table I:** Characteristics of AWs and healthy control subjects

Variable	AWs (n = 27)	Control subjects (n = 70)	*P* value
Child			
Sex			
Male	13 (48%)	32 (46%)	.829
Female	14 (52%)	38 (54%)	
Breast-feeding			
No	1 (4%)	1 (1%)	.494
Yes	26 (96%)	69 (89%)	
Period breast-feeding (mo)			
Median (range)	13 (4-20)	12 (3-24)	.510
Type of delivery			
Vaginal	17 (63%)	59 (84%)	**.022**
Caesarean	10 (37%)	11 (16%)	
Birth order			
First	9 (33%)	23 (33%)	.964
Second to third	11 (41%)	27 (39%)	
≥Fourth	7 (26%)	20 (28%)	
Household			
Maternal educational status			
Illiterate	1 (4%)	7 (10%)	.119
Primary complete	21 (78%)	41 (59%)	
Secondary complete	5 (18%)	22 (31%)	
Socioeconomic level			
Low	10 (37%)	26 (37%)	.994
Medium	9 (33%)	24 (34%)	
High	8 (30%)	20 (29%)	
Agricultural activities			
No	13 (48%)	34 (49%)	.970
Yes	14 (52%)	36 (51%)	
Potable water			
No	15 (56%)	52 (74%)	.074
Yes	12 (44%)	18 (26%)	
Bathroom			
Latrine/field	18 (67%)	44 (63%)	.992
WC	9 (33%)	26 (37%)	
Antibiotics and infections			
Antibiotics			
Maternal antibiotics in pregnancy			
No	9 (33%)	35 (50%)	.139
Yes	18 (67%)	35 (50%)	
Period (d)			
Median (IQR)	10 (3-21)	7 (1-48)	.113
During first year of life (courses)			
0	14 (52%)	41 (59%)	.196
1	11 (41%)	17 (24%)	
2-4	2 (7%)	12 (17%)	
Infections during first year			
Acute respiratory (episodes)			
0	0 (0%)	2 (3%)	**.011**
1	3 (26%)	35 (50%)	
2	11 (41%)	27 (38%)	
3-5	9 (33%)	6 (9%)	
Acute diarrhea			
No	13 (48%)	33 (47%)	.929
Yes	14 (52%)	37 (53)	

Text in boldface denotes statistical significance (*P* < .05). *IQR*, Interquartile range.

### Bacterial and fungal alterations associated with AW phenotype

**Fig E2:**
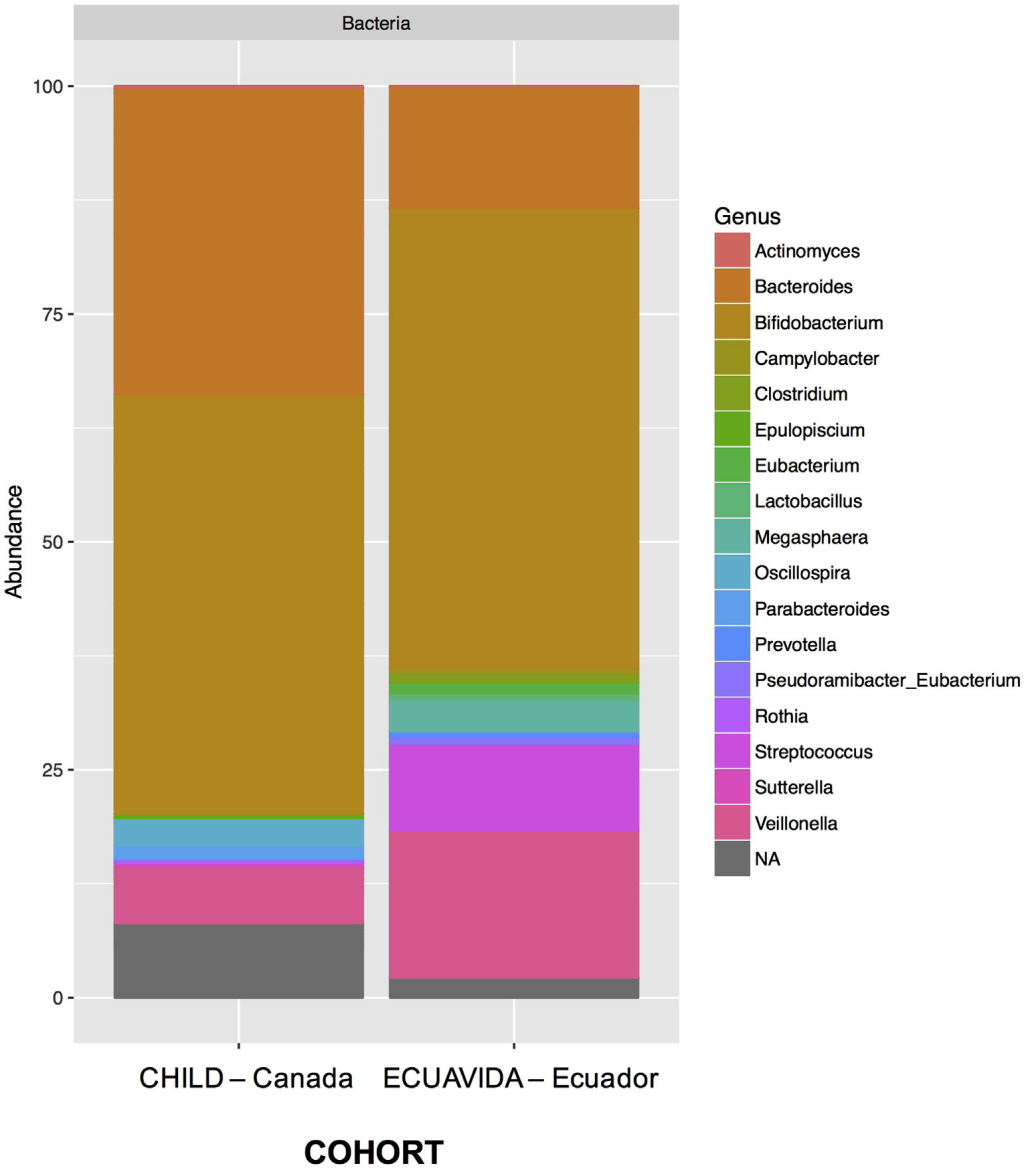
Relative abundance of the 100 most abundant bacterial OTUs in feces of Canadian versus Ecuadorian 3-month-old infants. *Rectangle colors* correspond to the bacterial genera in the legend. *Rectangles* represent merged OTUs belonging to the same genus. *NA*, OTUs without genus-level taxonomic assignment.

**Fig E3:**
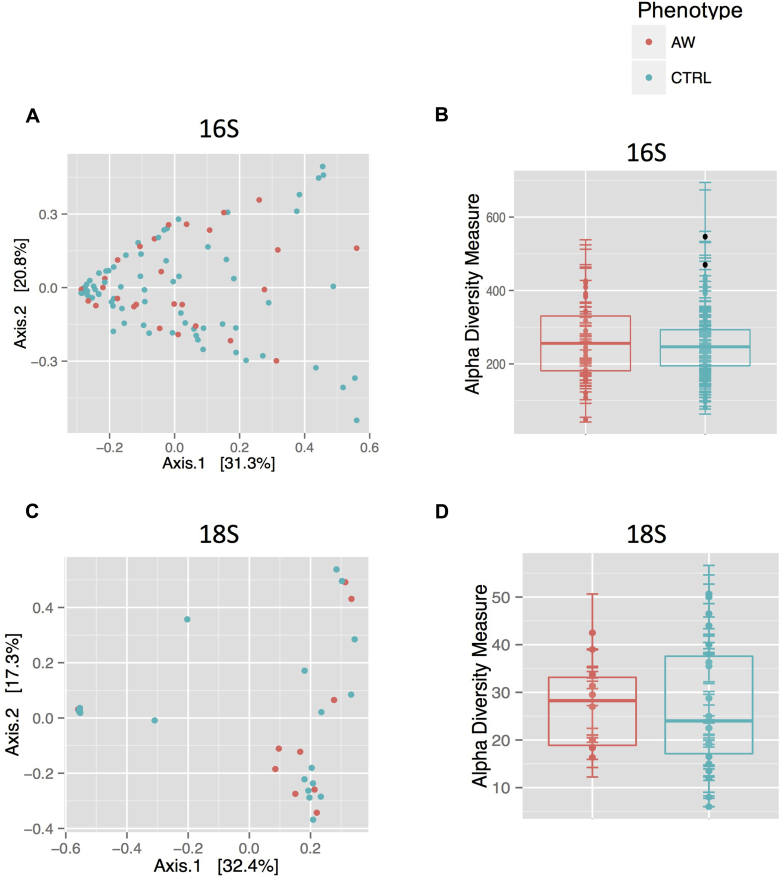
Both α (**A** and **C**; Chao1 index) and β (**B** and **D**; principal coordinates analysis [Bray-Curtis]) bacterial (Fig E3, *A* and *B*) and fungal (Fig E3, *C* and *D*) diversity in fecal samples from 3-month-old infants who presented with atopic wheeze at 5 years *(AW)* versus healthy control subjects *(CTRL)*. Sequencing results from all 16S samples and from 33 18S samples (control subjects, 23; AWs, 10) were retained after applying a cutoff of 1000 sequences per sample. The atopic wheeze phenotype did not explain significant changes in these measurements (*P* = .323 and .676 [Mann-Whitney test and permutational multivariate ANOVA] for Fig E3, *A* and *C*, respectively).

As expected, the gut microbiome between 3-month-olds in the Canadian CHILD Study and ECUAVIDA study differed substantially (see Fig E2 in this article's Online Repository at www.jacionline.org). In line with what we reported previously in Canadian infants, the AW phenotype did not explain any significant changes in α or β bacterial diversity (Mann-Whitney and permutational multivariate ANOVA, respectively; see Fig E3, *A* and *B*, in this article's Online Repository at www.jacionline.org). In addition to the bacterial profile, we also sequenced the V4 region of the 18S rRNA gene to obtain a profile of the intestinal eukaryotic microbiome in these infants. After subtracting plant and human sequences, our analysis found only sequences of fungal origin in the fecal microbiome of these 3-month-old babies. In this data set many samples had fewer than 1000 sequences per sample after applying filtering steps, which prompted their removal from the data set. Thus only 10 AW and 22 control samples were retained for statistical analysis of the 18S data set. However, AWs and control subjects used for fungal sequence analysis were reasonably representative of the original case and control samples (see Table E2 in this article's Online Repository at www.jacionline.org).

**Fig 1:**
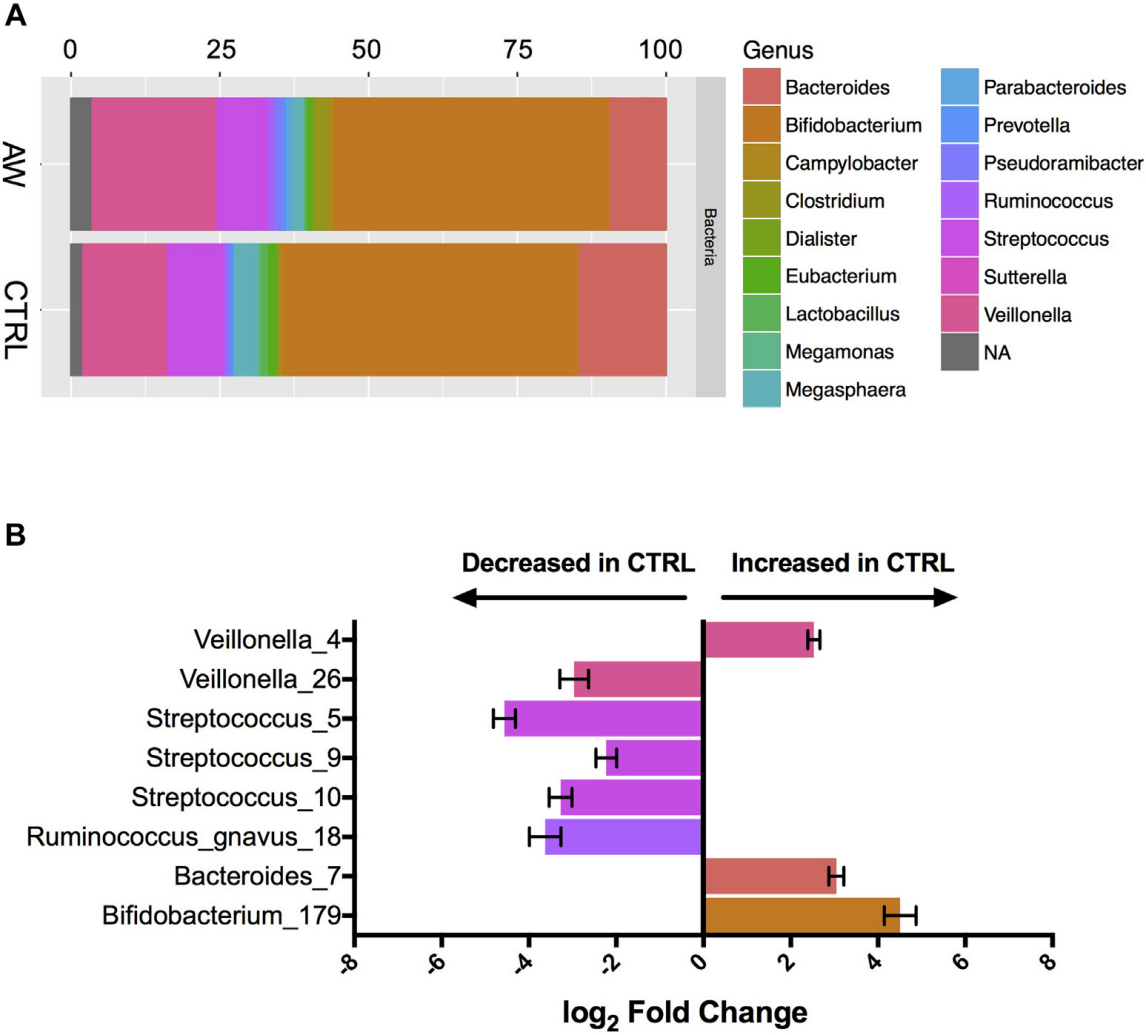
Bacterial dysbiosis associated with development of atopic wheeze. **A,** Relative abundance of bacterial genera within the top 100 OTUs among the 2 phenotypes at 3 months. Rectangle colors correspond to the bacterial genera in the legend. *Rectangles* represent merged OTUs belonging to the same genus. *NA*, OTUs without genus-level taxonomic assignment. **B,** Log fold change of OTUs that are significantly (false discovery rate < 0.05) abundant between AWs and control subjects calculated by using DESeq2. *Error bars* denote SDs. Taxonomic identification and OTU numbers are denoted on the *y-axis*.

**Fig 2:**
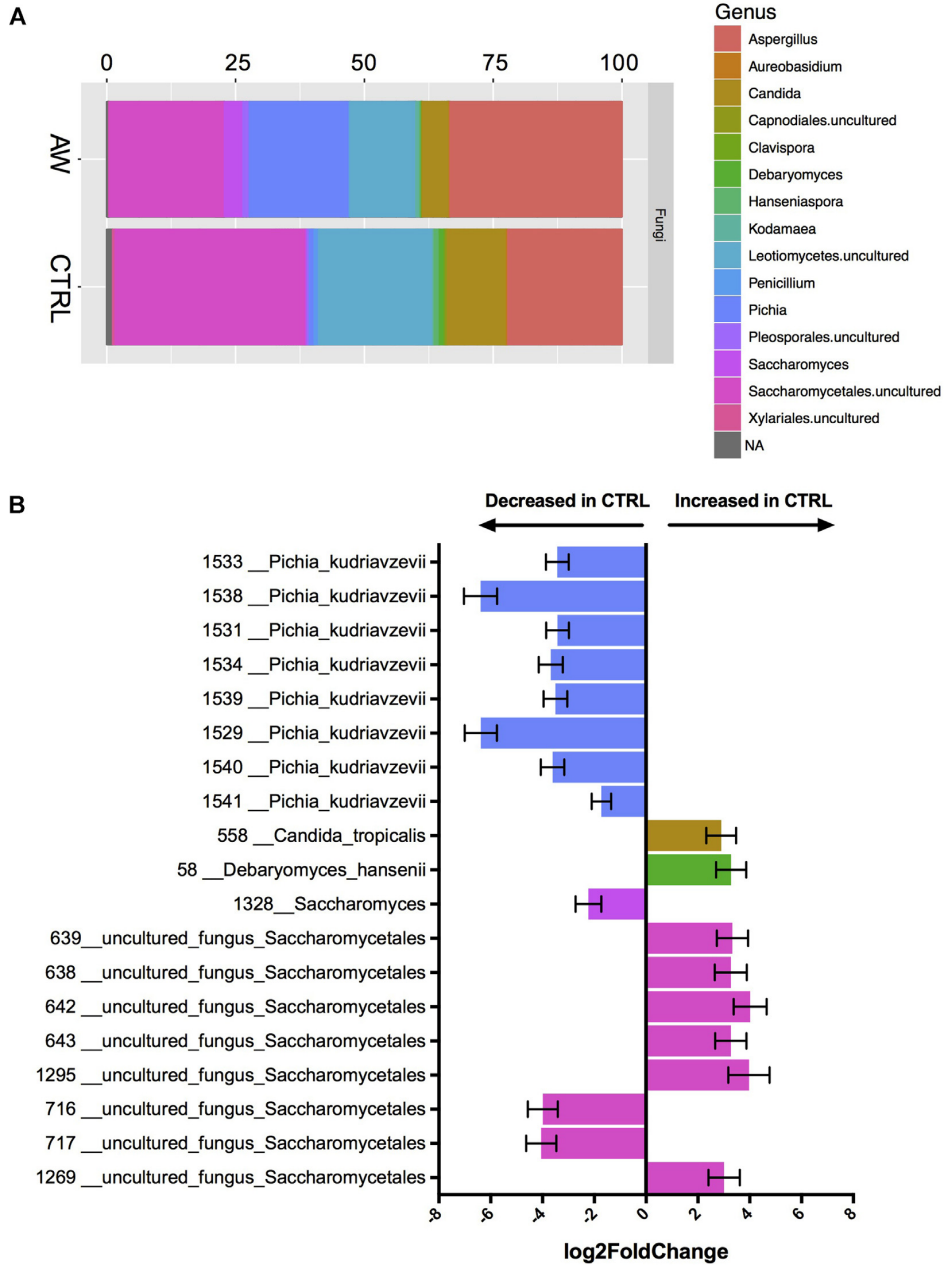
Fungal dysbiosis associated with development of atopic wheeze. **A,** Relative abundance of fungal genera within the top 100 OTUs among the 2 phenotypes at 3 months. Rectangle colors correspond to fungal genera in the legend. *Rectangles* represent merged OTUs belonging to the same genus or higher taxa if genus information was not obtained in the analysis. *NA*, OTUs without genus-level taxonomic assignment. **B,** Log fold change of OTUs significantly (false discovery rate < 0.05) abundant between AWs and control subjects calculated by using DESeq2. *Error bars* denote SDs. Taxonomic identification and OTU numbers are denoted on the *y-axis*.

**Fig E4:**
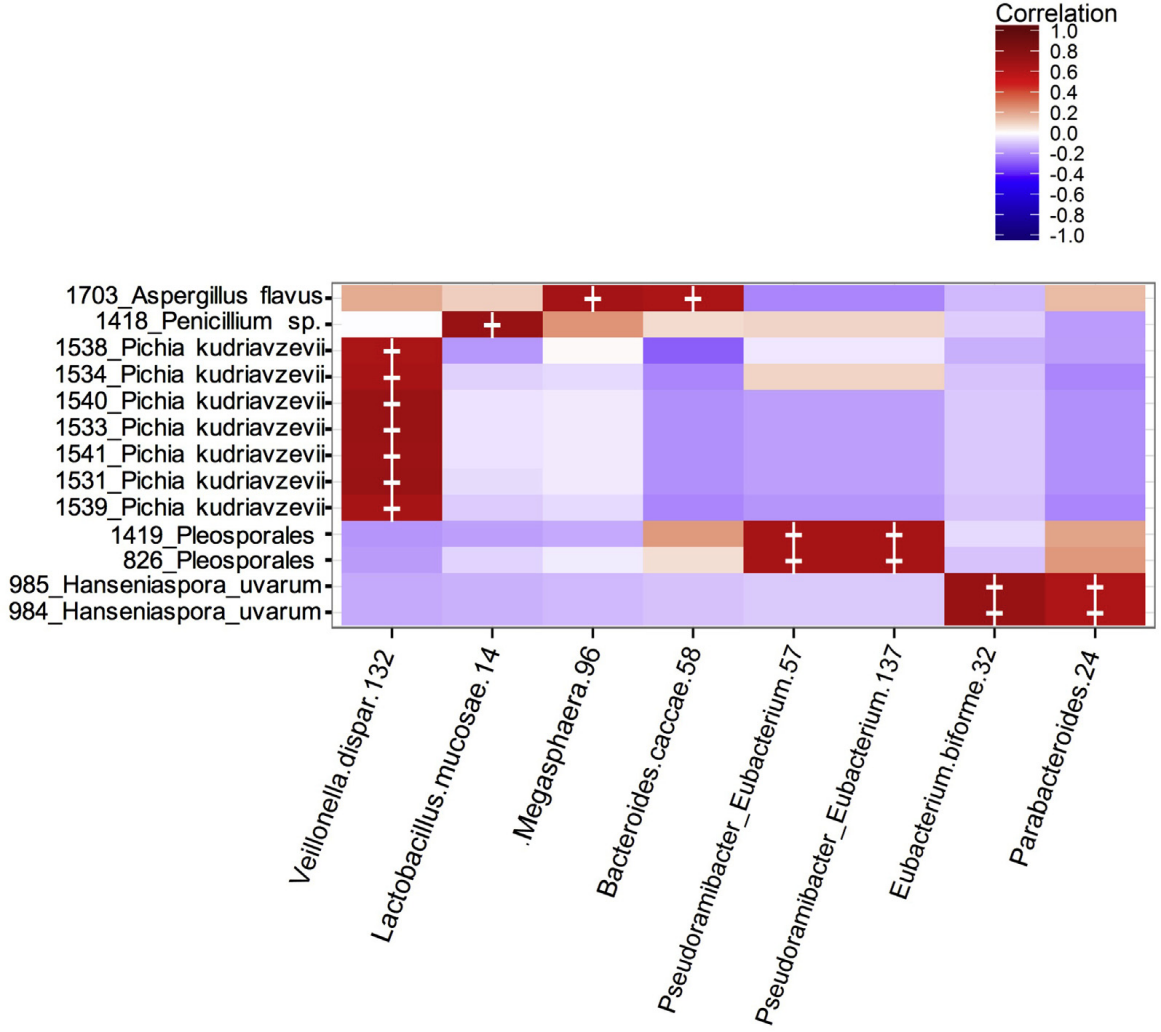
Heat map of biweight correlations between the top 100 bacterial *(x-axis)* and top 100 fungal *(y-axis)* OTUs in 33 fecal samples (control subjects, 23; AWs, 10) from 3-month-old infants recruited in the ECUAVIDA cohort study. Significant correlation values are denoted with a *cross* (*P* < .05, false discovery rate).

Similar to the 16S analysis, no significant differences were found in fungal α or β diversity between infants with atopic wheeze and control subjects (see Fig E3, *C* and *D*). Despite the lack of broad microbial community shifts between AWs and control subjects, several OTUs of bacteria (Fig 1) and especially fungi (Fig 2) were found to be differentially abundant by using DESeq2, a method of differential analysis of high-throughput sequencing data based on a model of negative binomial distribution. Among the observed bacterial alterations were 2 OTUs identified as *Veillonella* species, 3 OTUs identified a *Streptococcus* species, 1 OTU identified as *Bacteroides* species, 1 OTU identified as *Ruminococcus gnavus*, and 1 OTU identified as *Bifidobacterium* species (DESeq2; *P* < .05, Wald test and false discovery rate). Interestingly, 5 of these OTUs were among the top 10 most abundant bacterial OTUs in the data set. Notably, more than twice number of fungal OTUs (19) were significantly different between AWs and control subjects compared with bacterial OTUs, with 7 of them identified as the yeast *P kudriavzevii* (Fig 2, *B*)*.* The larger differences in the fungal versus bacterial microbiome were also evident in relative abundance plots of the top 100 most abundant bacterial and fungal taxa, organized by genus (Figs 1, *A*, and 2, *A*). Additionally, we found several correlations between bacterial and fungal OTUs, suggesting potential ecologic synergistic and antagonistic associations occurring in the infant gut (see Fig E4 in this article's Online Repository at www.jacionline.org).

Given extensive metadata collected in this cohort, we captured the effect of the gut microbiome on the atopic wheeze phenotype (used as a surrogate for asthma risk) while controlling for potential confounding using MaAsLin.^43^ MaAsLin is a multivariate linear modeling tool with boosting that tests for associations between specific microbial taxa and continuous and/or Boolean metadata. In microbiome studies certain variables, such as diet, age, and sample origin, can have large effects on microbiome composition. Thus it is important to subtract their influence from the potential effect caused by other metadata in the study, such as the phenotype. With MaAsLin, we found a significant association between most of the differential taxa obtained through DESeq2 analysis and atopic wheeze, including the bacterial genera *Bifidobacterium*, *Streptococcus*, and *Veillonella*, as well as the fungi *P kudriavzevii*, *Saccharomyces*, and Saccharomycetales (see Tables E3 and E4 in this article's Online Repository at www.jacionline.org), supporting the strong association between these microbial changes and the phenotype. By using both statistical approaches, it was evident that fungal alterations were more marked than bacterial dysbiosis.

**Fig 3:**
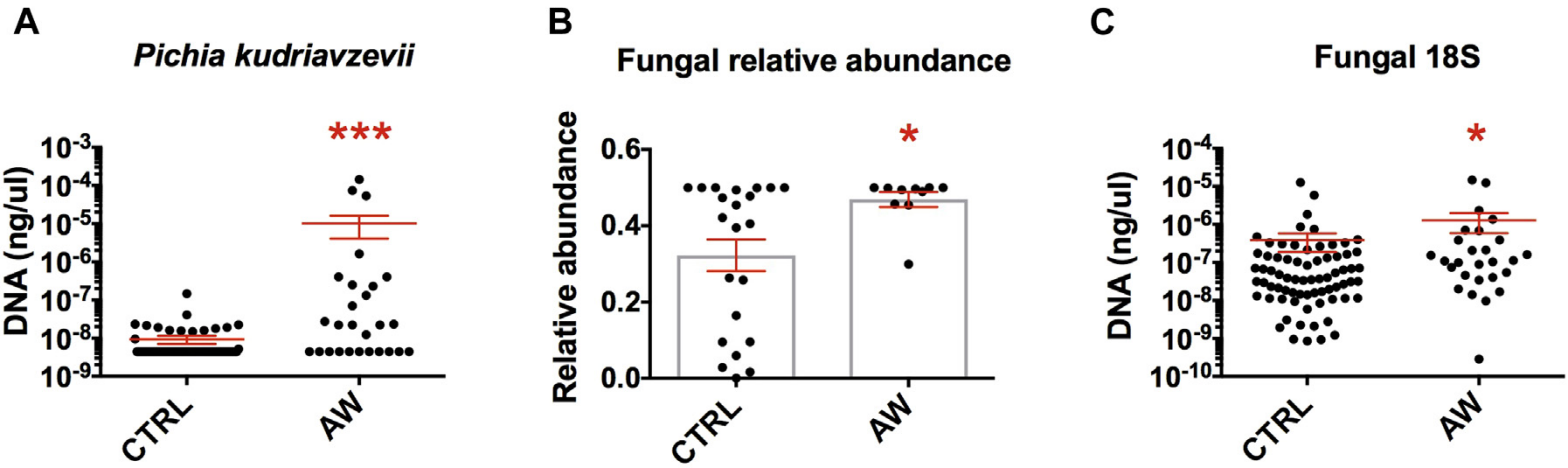
Fungus-specific qPCR and Illumina sequence data. **A,** qPCR quantification (standard curve method) of *P kudriavzevii* in feces of 3-month-old infants from the ECUAVIDA study (control subjects, 68; AWs, 27). **B,** Relative abundance of fungal reads over the total number of eukaryotic reads from 18S data set. Only samples with more than 1000 sequences after filtering were included in the 18S high-throughput sequencing analysis (control subjects, 23; AWs, 10). **C,** qPCR quantification (standard curve method) of fungus-specific 18S copies in feces of 3-month-old infants from the ECUAVIDA study (control subjects, 68; AWs, 27). In Fig 3, *A* and *C*, graphs are based on a logarithmic scale. All graphs show Mann-Whitney tests: *P < .05 and ***P < .001.

**Fig E5:**
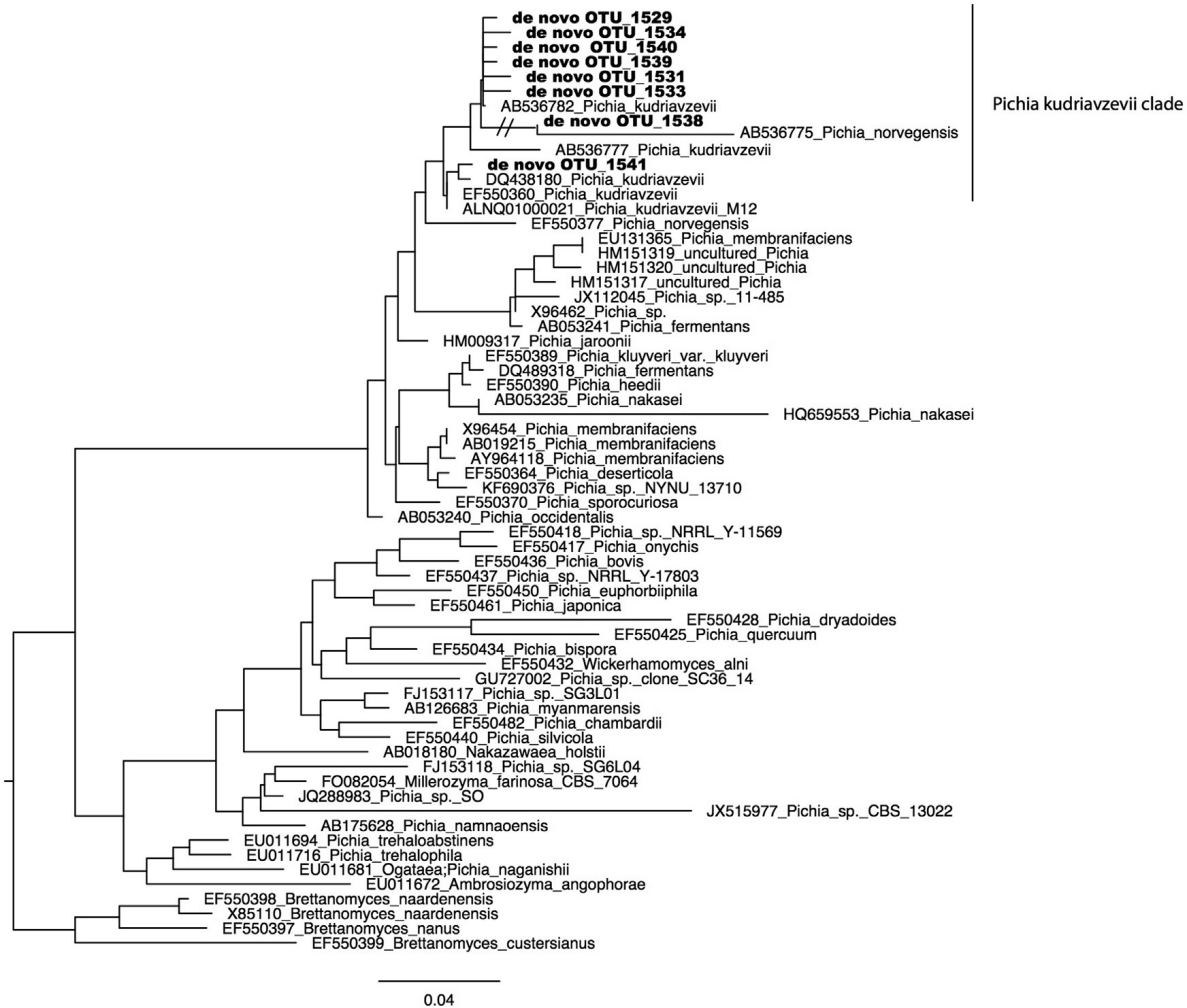
Phylogenetic placement of *P kudriavzevii* OTUs within *Pichia* species sequences from the SILVA 123 reference database. 18S ribosomal DNA sequences labeled as *Pichia* species or Pichiaceae in the 99% SILVA reference database were aligned and then used to construct a reference tree with RAxML. OTUs from this study assigned to *Pichia* species were placed in this tree with the RAxML evolutionary placement algorithm. All OTUs cluster within a clade of *P kudriavzevii*.

Because of the strength of the association between the yeast *P kudriavzevii* and atopic wheeze risk, we validated the sequencing result using maximum likelihood to place individual sequences assigned as *P kudriavzevii* into a phylogenetic tree of all sequences assigned to *Pichia* species yeast in the reference database and found that all of the OTUs fell within the *P kudriavzevii* clade (see Fig E5 in this article's Online Repository at www.jacionline.org). We further validated this result by designing and optimizing specific primers for this yeast. Using qPCR on all AW and control samples, we confirmed the increased prevalence of *Pichia* species in the stool of 3-month-old babies who went on to have atopic wheeze in this Ecuadorian cohort (Fig 3, *A*). Although there is overlap in values between groups, almost a third of child AWs (8/27) had a higher abundance of *P kudriavzevii* than the highest abundance among control subjects (*P* = .001).

In addition, we found that AW samples contained a higher proportion of fungal reads over all eukaryotic reads (Fig 3, *B*). To confirm this bioinformatic observation, we quantified total fungal 18S rRNA gene copies using qPCR in all samples. Notably, we found a small yet significant increase in 18S rRNA gene copies in the AW samples (Fig 3, *C*), suggesting that fungi are overrepresented in the gut microbiome of babies who go on to have atopic wheeze.

### Functional microbiome alterations in babies who had atopic wheeze

**Fig E6:**
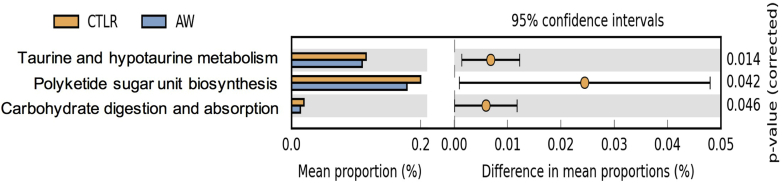
PICRUSt-predicted Kyoto Encyclopedia of Genes and Genomes functional categories with significant differences in relative abundance between control subjects and AWs (Welch *t* test, *q* values are shown in figure).

To determine the functional potential of the bacterial dysbiosis detected by using 16S sequencing, we predicted the microbiome metagenome using PICRUSt.^32^ PICRUSt is a computational method that predicts functional composition based on the 16S rRNA gene content and copy number and reference bacterial genomes. By comparing the predicted genetic composition between the 2 groups, we found taurine and hypotaurine metabolism, polyketide sugar unit biosynthesis, and carbohydrate digestion and absorption pathways were decreased in AWs (Welch *t* test, see Fig E6 in this article's Online Repository at www.jacionline.org).

**Fig 4:**
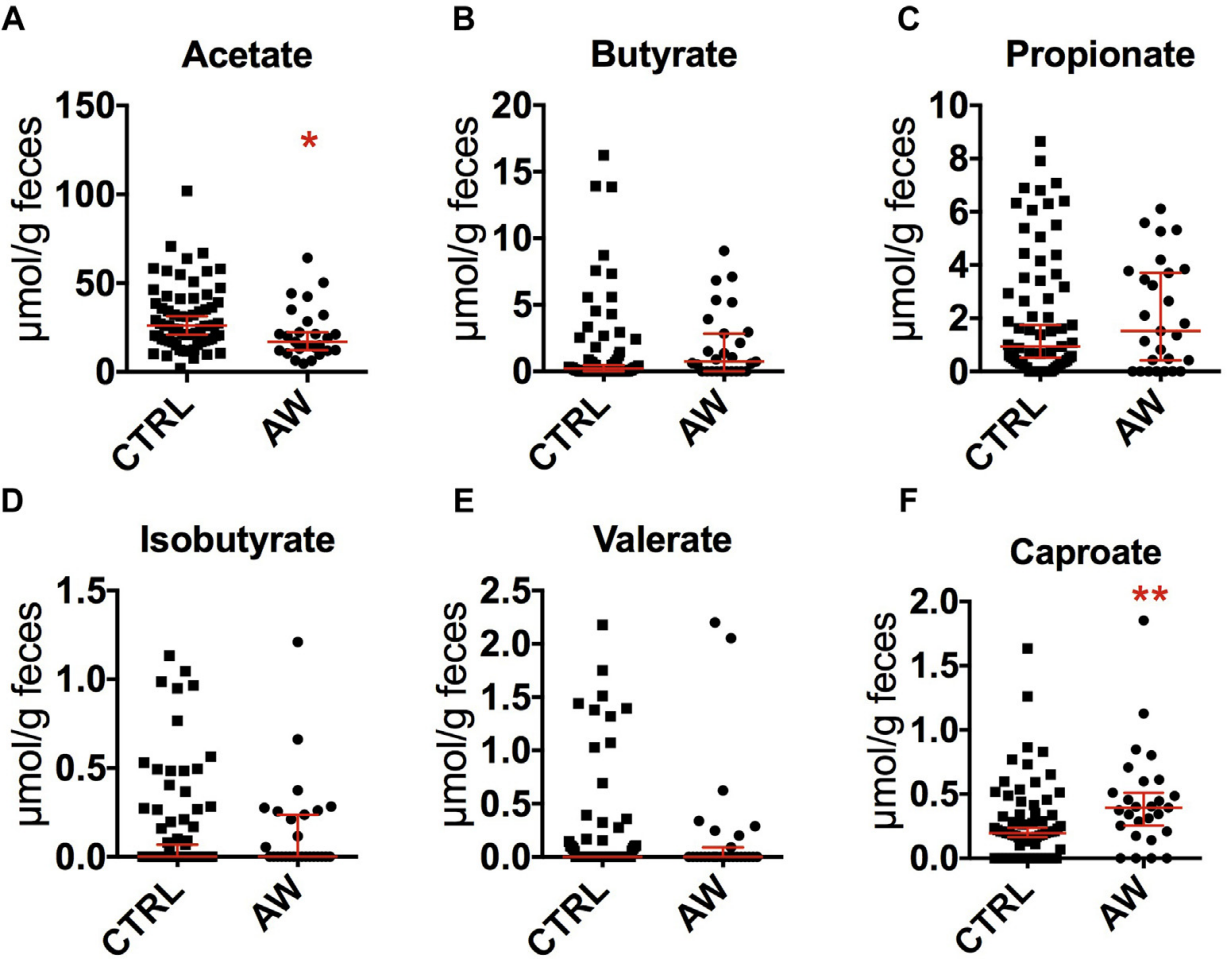
Concentration of the 6 most abundant SCFAs in feces of infants with atopic wheeze and control infants at 3 months of age measured by using gas chromatography and normalized to feces wet weight. Acetate **(A)**, butyrate **(B)**, propionate **(C)**, isobutyrate **(D)**, valerate **(E)**, and caproate **(F)**. (AWs, 27; control subjects, 70; *P < .05 and **P < .01, Mann-Whitney test).

We investigated further the predicted change in bacterial carbohydrate metabolism by measuring SCFA concentrations in feces. Similar to what we found in babies from the CHILD study, there was a significant decrease in acetate concentrations in the stool of babies in the ECUAVIDA study who went on to have atopic wheeze (Fig 4, *A*), suggesting similar gut bacterial fermentation patterns between the 2 populations. In addition, we detected an increase in caproate levels associated with the AW phenotype in this cohort (Fig 4, *F*).

## Discussion

In the present study we explored the association between gut bacterial and fungal dysbiosis in infancy and the development of atopic wheeze in later childhood in a representative sample of an Ecuadorian birth cohort (ECUAVIDA), which has studied a population living in a rural district in a tropical nonindustrialized country. This represents a distinct environment from all previous studies of the effects of the microbiome on asthma development done in developed settings. In agreement with previous analyses from Canadian^7^, ^8^ and US^11^ birth cohorts, our findings provide further confirmation of the presence of a microbial signature in the first 100 days of life associated with pediatric risk. A direct comparison between these new findings and the dysbiosis present in the Canadian CHILD study is not entirely possible because of differences in study design. In the CHILD study atopic wheeze was defined at 1 year of age, whereas this study measured atopic wheezing at 5 years as a surrogate for asthma risk. However, the observed differences in gut dysbiosis associated with the risk of atopic wheezing in both cohorts could also originate from differences in gut microbial composition, given that the microbiome of babies in ECUAVIDA differed substantially from that in age-matched Canadian babies in the CHILD study. As has been shown previously,^44^ modernization processes (or westernization) strongly affect the human gut microbiome. Although this study was not designed to study the factors that lead to such differences, the humid warm weather in this region and less hygienic conditions are likely to increase exposures to microbes, including those that cause childhood infectious diseases, which might affect health and modify the infant microbiome.^14^, ^45^

The differences in the gut microbiome detected in this cohort yielded important information. First, the microbial signature associated with atopic wheeze in these infants was more pronounced than in the Canadian cohort. In addition, except for *Veillonella* species, the bacterial dysbiosis associated with atopic wheezing in this cohort involved different taxa to those detected in the Canadian study. Despite major compositional differences associated with atopic wheezing between the Canadian and Ecuadorian cohorts, we found important metabolic similarities between the 2 cohorts. Notably, a decrease in fecal acetate in atopic infants with wheeze was a common finding in the 2 populations, suggesting that alterations in fermentative patterns that led to a decrease in acetate production might be a common feature associated with atopic wheezing. A reduction in carbohydrate metabolism predicted by PICRUSt also supports the association between SCFA production and atopic wheeze. Acetate,^46^ butyrate,^47^ and propionate^46^ have been shown to stimulate regulatory T-cell proliferation and attenuate allergy-associated T_H_2 immune responses.^48^ Given our data in 2 very different cohorts, fecal acetate levels reductions should be studied further in larger longitudinal cohorts as a possible biomarker of asthma risk in young babies.

Prompted by the results from a recent study in US infants,^11^ we profiled the eukaryotic microbiome of the Ecuadorian babies using the 18S rRNA marker gene and a custom-annotated taxonomic database. Interestingly, after removing mammalian and plant-derived sequences, only fungal sequences remained in the data set. Given that we have successfully detected other eukaryotic commensals using the same methodological approach in adult human cohorts (data not shown), this finding indicates that the eukaryotic microbiome in early life consists mainly of fungi, at least in this population. Filamentous fungi and yeasts are common members of the normal gut microbiome, although in a much smaller proportion than bacteria.^49^ However, it has been reported that during the first 3 months of life, they are present at a significantly higher diversity than later in life.^11^ Furthermore, their role in immune regulation and asthma development has been documented previously in relevant models of murine lung inflammation. In particular, modulation of the mycobiome has been shown to increase the severity of murine lung inflammation.^50^ Moreover, antibiotic-induced blooms of *Candida* species were associated with increased experimental murine lung inflammation.^51^, ^52^ This effect has been shown to be mediated by *Candida* species–produced prostaglandin E_2_,^53^, ^54^, ^55^ an arachidonic acid derivative known play an important role in orchestrating immune interactions relevant in asthmatic patients.

Among the 19 fungal OTUs associated with the AW phenotype (twice as many as the differential bacterial OTUs detected), 7 of them corresponded to the yeast *P kudriavzevii*. The robustness of the statistical association between this yeast and the AW phenotype prompted us to further validate and confirm its taxonomic identity bioinformatically and through qPCR, especially given the reduced sample size used in the 18S sequencing analysis. We note that there is sufficient variation in this 18S region to identify *Pichia* species and distinguish between relatives.^56^ *P kudriavzevii* has been described as a commensal member of the gut mycobiome,^57^ and to our knowledge, this is the first report of this species implicated in gut microbial dysbiosis associated with atopic disease. Notably, qPCR analysis in all the samples detected not only an increase in *P kudriavzevii* but also an increase in fungi-specific 18S gene copies, suggesting, also for the first time, that fungal overrepresentation in the infant gut microbiome is associated positively with the development of atopic wheeze. However, given the size of the sample studied and the small but significant differences detected in fungal gene copies and reads, caution should be taken when extrapolating these findings to other populations. These findings should be replicated in other human cohorts before considering gut fungal overrepresentation, as associated with asthma risk.

Evidently, fungal dysbiosis was more pronounced than bacterial dysbiosis in this study. Thus the role of *P kudriavzevii* and other yeasts should be explored in mechanistic studies using animal models. In addition to being important members of the gut microbiome, fungal species can experience an ecological advantage over bacteria after antibiotic treatment, a very strong risk factor associated with asthma development in most human cohorts.^3^

Collectively, these data support our previously proposed concept of a critical window in early life during which microbial dysbiosis can be detected in strong association with a subsequent allergic phenotype predictive of asthma.^9^ The nature of this dysbiosis appears to reflect the microbiome composition in a given population, prompting additional analysis in prospective studies around the world and inclusion of fungal analyses in all microbial surveys. Optimized biomarker studies of the microbial taxa and the metabolites involved in regional asthma-associated dysbiosis could help identify infants at risk of asthma before symptoms occur, as well as provide a scientific rationale for future therapeutic strategies aimed at restoring an altered infant gut microbiome.

**Clinical implications**

Our findings support the importance of early-life infant gut microbial dysbiosis in the development of atopic wheeze and could be used for the development of novel asthma primary prevention strategies through modification of the microbiome in early life.

## Figures and Tables

**Table E1:** Characteristics of AWs at 5 years comparing those included in the analysis with those excluded from the analysis

Variable	Included (n = 27)	Excluded (n = 32)	*P* value
Child			
Sex			
Male	13 (48%)	21 (66%)	.176
Female	14 (52%)	11 (34%)	
Breast-feeding			
No	1 (4%)	0 (0%)	.280
Yes	26 (96%)	32 (100%)	
Period breast-feeding (mo)			
Median (range)	13 (4-20)	13 (10-18)	.948
Type of delivery			
Vaginal	17 (63%)	23 (72%)	.465
Caesarean	10 (37%)	9 (28%)	
Birth order			
First	9 (33%)	6 (19%)	.230
Second to third	11 (41%)	14 (44%)	
≥Fourth	7 (26%)	12 (37%)	
Household			
Maternal educational status			
Illiterate	1 (4%)	4 (12%)	.083
Primary complete	21 (78%)	16 (50%)	
Secondary complete	5 (18%)	12 (38%)	
Socioeconomic level			
Low	10 (37%)	9 (28%)	.529
Medium	9 (33%)	9 (28%)	
High	8 (30%)	14 (44%)	
Agricultural activities			
No	13 (48%)	15 (47%)	.922
Yes	14 (52%)	17 (53%)	
Potable water			
No	15 (56%)	21 (66%)	.429
Yes	12 (44%)	11 (34%)	
Bathroom			
Latrine/field	17 (63%)	23 (72%)	.465
WC	10 (37%)	9 (28%)	
Antibiotics and infections			
Antibiotics			
Maternal antibiotics in pregnancy			
No	9 (33%)	14 (44%)	.414
Yes	18 (67%)	18 (56%)	
Period (d)			
Median (IQR)	10 (3-21)	8 (5-10)	.101
During first year of life (courses)			
0	14 (52%)	18 (56%)	.455
1	11 (41%)	9 (28%)	
2-4	2 (7%)	5 (16%)	
Infections during first year			
Acute respiratory (episodes)			
0	0 (0%)	2 (6%)	.597
1	3 (26%)	9 (28%)	
2	11 (41%)	12 (38%)	
3-5	9 (33%)	9 (28%)	
Acute diarrhea			
No	13 (48%)	12 (38%)	.410
Yes	14 (52%)	20 (62%)	

Inclusion in the analysis was based on having a stool sample collected at 3 months of age.

*IQR*, Interquartile range.

**Table E2:** Characteristics of study subjects in a reduced cohort for whom fungal sequencing data were analyzed

Variable	AWs (n = 10)	Control subjects (n = 22)	*P* value
Child			
Sex			
Male	7 (70%)	11 (50%)	.253
Female	3 (30%)	11 (50%)	
Breast-feeding			
No	0 (0%)	0 (0%)	—
Yes	10 (100%)	22 (100%)	
Period breast-feeding (mo)			
Median (range)	14 (9-16)	13 (12-16)	.962
Type of delivery			
Vaginal	7 (70%)	18 (82%)	.376
Caesarean	3 (30%)	4 (18%)	
Birth order			
First	5 (50%)	7 (32%)	.412
Second to third	2 (20%)	9 (41%)	
≥Fourth	3 (30%)	6 (27%)	
Household			
Maternal educational status			
Illiterate	0 (0%)	1 (5%)	.358
Primary complete	9 (90%)	14 (64%)	
Secondary complete	1 (10%)	7 (32%)	
Socioeconomic level			
Low	2 (20%)	10 (45%)	.318
Medium	3 (30%)	7 (32%)	
High	5 (50%)	5 (23%)	
Agricultural activities			
No	6 (60%)	10 (45%)	.352
Yes	4 (40%)	12 (55%)	
Potable water			
No	4 (40%)	13 (59%)	.267
Yes	6 (60%)	9 (41%)	
Bathroom			
Latrine/field	8 (80%)	16 (73%)	.512
WC	2 (20%)	6 (27%)	
Antibiotics and infections			
Antibiotics			
Maternal antibiotics in pregnancy			
No	3 (30%)	13 (59%)	.126
Yes	7 (70%)	9 (41%)	
Period (d)			
Median (IQR)	7 (0-9)	0 (0-5)	.109
During first year of life (courses)			
0	7 (70%)	12 (55%)	.631
1	3 (30%)	8 (36%)	
2-4	0 (0%)	3 (13%)	
Infections during first year			
Acute respiratory (episodes)			
0	0 (0%)	1 (5%)	.12
1	3 (30%)	14 (63%)	
2	4 (40%)	6 (27%)	
3-5	3 (30%)	1 (5%)	
Acute diarrhea			
No	5 (50%)	10 (45%)	.81
Yes	5 (50%)	12 (55%)	

*IQR*, Interquartile range.

**Table E3:** Differentially abundant microbial OTUs (false discovery rate < 0.05) between AWs and control subjects calculated by using DESeq2

Variable	Feature	log_2_ Fold change	*P* value (adjusted)	Effect	95% CI
Bacterial features					
Phenotype	Otu4_*Veillonella*	−2.5303623	3.98E-11	Increased	−2.6510012 to −2.4097234
Phenotype	Otu5_*Streptococcus*	4.56211499	3.98E-11	Decreased	4.34260055 to 4.78162944
Phenotype	Otu7_*Bacteroides*	−3.0482054	7.12E-11	Increased	−3.1976966 to −2.8987142
Phenotype	Otu9_*Streptococcus*	2.2278997	.00400262	Decreased	2.01960114 to 2.43619825
Phenotype	Otu010_*Streptococcus*	3.27520397	2.04E-05	Decreased	3.04766741 to 3.50274053
Phenotype	Otu018_*Ruminococcus_gnavus*	3.62669523	.00162925	Decreased	3.30974875 to 3.94364171
**Phenotype**	**Otu026_*Veillonella***	**2.96323589**	**.00561369**	**Decreased**	**2.67680938 to 3.2496624**
**Phenotype**	**Otu179_*Bifidobacterium***	**−4.510034**	**2.25E-05**	**Increased**	**−4.8273551 to −4.1927128**
Fungal features					
Phenotype	58 __*Debaryomyces_hansenii*	3.28100437	5.64E-05	Increased	2.77607299 to 3.78593574
Phenotype	558 __*Candida_tropicalis*	2.89814469	.00031816	Increased	2.39918176 to 3.39710762
Phenotype	638 __uncultured_fungus_Saccharomycetales	3.26985979	.00015227	Increased	2.73474031 to 3.80497926
Phenotype	639__*Saccharomycetes*	3.33253614	8.21E-05	Increased	2.80805337 to 3.85701891
Phenotype	642 __uncultured_fungus_Saccharomycetales	4.01492817	6.51E-06	Increased	3.46305197 to 4.56680436
Phenotype	643 __uncultured_fungus_Saccharomycetales	3.27079498	.0001125	Increased	2.74586052 to 3.79572945
**Phenotype**	**716 __uncultured_fungus_Saccharomycetales**	**−3.9866517**	**7.64E-07**	**Decreased**	**−4.4864069 to −3.4868965**
**Phenotype**	**717 __uncultured_fungus_Saccharomycetales**	**−4.048536**	**5.97E-07**	**Decreased**	**−4.5511721 to −3.5458998**
Phenotype	1269 __uncultured_fungus_Saccharomycetales	3.01162348	.000382	Increased	2.48614246 to 3.5371045
Phenotype	1295 __uncultured_fungus_Saccharomycetales	3.97293655	.00036181	Increased	3.28249384 to 4.66337926
Phenotype	1328__*Saccharomyces*	−2.2265938	.00135391	Decreased	−2.6572829 to −1.7959046
Phenotype	1529 __*Pichia_kudriavzevii*	−6.3822352	2.53E-13	Decreased	−6.9228509 to −5.8416194
**Phenotype**	**1531 __*Pichia_kudriavzevii***	**−3.421152**	**2.82E-08**	**Decreased**	**−3.8030808 to −3.0392231**
**Phenotype**	**1533 __*Pichia_kudriavzevii***	**−3.4287207**	**2.23E-08**	**Decreased**	**−3.8086674 to −3.048774**
**Phenotype**	**1534 __*Pichia_kudriavzevii***	**−3.6780058**	**1.55E-08**	**Decreased**	**−4.0810328 to −3.2749788**
**Phenotype**	**1538 __*Pichia_kudriavzevii***	**−6.3906936**	**9.03E-13**	**Decreased**	**−6.9451013 to −5.8362859**
**Phenotype**	**1539 __*Pichia_kudriavzevii***	**−3.5008997**	**3.85E-08**	**Decreased**	**−3.8956113 to −3.1061881**
**Phenotype**	**1540 __*Pichia_kudriavzevii***	**−3.61174**	**1.14E-08**	**Decreased**	**−4.0039118 to −3.2195682**
**Phenotype**	**1541 __*Pichia_kudriavzevii***	**−1.7264597**	**.00096672**	**Decreased**	**−2.0507199 to −1.4021995**

*P* values are corrected for false discovery rate. OTUs in boldface remained statistically significant after controlling for confounding (Table E4).

**Table E4:** Microbial taxa associated with atopic wheezing corrected for major confounding effects known to affect microbiome composition by using MaAsLin

Variable	Feature	Coefficient	*P* value (adjusted)	*q* Value	Effect	95% CI
Bacterial features						
**Phenotype**	***Bifidobacterium*_179**	**0.02299582**	**1.05E-12**	**8.64E-11**	**Increased**	**0.02169825 to 0.0242934**
Phenotype	*Veillonella*_Dispar_132	−0.0092217	.00254641	0.10440262	Decreased	−0.0106249 to −0.0078185
Phenotype	*Veillonella*_86	−0.007847	.02938598	0.6277335	Decreased	−0.0094878 to −0.0062061
**Phenotype**	***Veillonella*_26**	**−0.0167265**	**.03077532**	**0.6277335**	**Decreased**	**−0.0201968 to −0.0132562**
Phenotype	*Streptococcus*_135	−0.0050144	.0444958	0.6277335	Decreased	−0.0071267 to −0.0029021
Phenotype	*Bifidobacterium*_103	0.00845423	.04751864	0.6277335	Increased	0.00639847 to 0.01050998
Fungal features						
**Phenotype**	***Pichia_Kudriavzevii*_1538**	**−0.2039807**	**.00325965**	**0.08131335**	**Decreased**	**−0.2268391 to 0.28743912**
**Phenotype**	***Pichia_Kudriavzevii*_1534**	**−0.035631**	**.00350364**	**0.08131335**	**Decreased**	**−0.0396671 to 0.05036711**
**Phenotype**	***Pichia_Kudriavzevii*_1531**	**−0.0358471**	**.00431312**	**0.08131335**	**Decreased**	**−0.040034 to 0.05113405**
**Phenotype**	***Pichia_Kudriavzevii*_1533**	**−0.0309382**	**.00496049**	**0.08131335**	**Decreased**	**−0.0346121 to 0.04435211**
**Phenotype**	***Pichia_Kudriavzevii*_1539**	**−0.0321617**	**.00555307**	**0.08131335**	**Decreased**	**−0.0360469 to 0.04634688**
**Phenotype**	***Pichia_Kudriavzevii*_1541**	**−0.0250888**	**.00638293**	**0.08131335**	**Decreased**	**−0.0281781 to 0.0363681**
**Phenotype**	***Pichia_Kudriavzevii*_1540**	**−0.0309761**	**.00694138**	**0.08131335**	**Decreased**	**−0.0348236 to 0.04502356**
Phenotype	*Saccharomyces*_1327	−0.1066187	.0088887	0.09110916	Decreased	−0.1204243 to 0.15702429
Phenotype	*Aspergillus_flavus*_1703	−0.2422061	.02612342	0.22469852	Decreased	−0.2799263 to 0.37992633
Phenotype	*Aspergillus_flavus*_1631	−0.2258779	.02740226	0.22469852	Decreased	−0.2615235 to 0.35602348
**Phenotype**	**Saccharomycetales_Uncultured_716**	**−0.1691154**	**.03545843**	**0.26432651**	**Decreased**	**−0.1853351 to 0.22833514**
**Phenotype**	**Saccharomycetales_Uncultured_717**	**−0.1835183**	**.04168681**	**0.26766654**	**Decreased**	**−0.1899307 to 0.20693072**
Phenotype	Leotiomycetes_Uncultured_1500	−0.0163321	.04646276	0.26766654	Decreased	−0.0223673 to 0.03836733

Direction of the correlation coefficient is in relation to the control group. *P* values are corrected for false discovery rate. Boldface represents the same OTUs identified as significant by using DESeq2 analysis (see Table E3).
